# Impact of the Salud Mesoamerica Initiative on delivery care choices in Guatemala, Honduras, and Nicaragua

**DOI:** 10.1186/s12884-021-04279-2

**Published:** 2022-01-03

**Authors:** Bernardo Hernandez, Katie Panhorst Harris, Casey K. Johanns, Erin B. Palmisano, Rebecca Cogen, Maximilian G. Thom, Emily Linebarger, Charbel El Bcheraoui, Aruna M. Kamath, Joseph Camarda, Diego Rios-Zertuche, María Paola Zúñiga-Brenes, Pedro Bernal-Lara, Danny Colombara, Alexandra Schaefer, Benito Salvatierra, Julio César Mateus, Isabel Casas, Giovanni Flores, Emma Iriarte, Ali H. Mokdad

**Affiliations:** 1 Institute for Health Metrics and Evaluation. Department of Health Metrics Sciences, University of Washington, Seattle, WA USA; 2 Institute for Health Metrics and Evaluation, University of Washington, Seattle, WA USA; 3 Robert Koch Institute, Berlin, Germany; 4 Department of Anesthesiology, University of Washington, Seattle, WA USA; 5 Salud Mesoamerica Initiative, Inter-American Development Bank, Washington, DC USA; 6 Salud Mesoamerica Initiative, Inter-American Development Bank, San Jose, Costa Rica; 7 Seattle & King County. Assessment, Policy Development, & Evaluation Unit, Seattle, WA USA; 8 Global Center for Integrated Health of Women, Adolescents, and Children, University of Washington, Seattle, WA USA; 9 Departamento de Salud, El Colegio de la Frontera Sur, San Cristóbal de las Casas, Chiapas Mexico; 10 Universidad del Valle, Cali, Colombia; 11 UNIMER, San Salvador, El Salvador

**Keywords:** Maternal and child health, Evaluation, Low-income countries

## Abstract

**Background:**

The Salud Mesoamérica Initiative (SMI) is a public-private collaboration aimed to improve maternal and child health conditions in the poorest populations of Mesoamerica through a results-based aid mechanism. We assess the impact of SMI on the staffing and availability of equipment and supplies for delivery care, the proportion of institutional deliveries, and the proportion of women who choose a facility other than the one closest to their locality of residence for delivery.

**Methods:**

We used a quasi-experimental design, including baseline and follow-up measurements between 2013 and 2018 in intervention and comparison areas of Guatemala, Nicaragua, and Honduras. We collected information on 8754 births linked to the health facility closest to the mother’s locality of residence and the facility where the delivery took place (if attended in a health facility). We fit difference-in-difference models, adjusting for women’s characteristics (age, parity, education), household characteristics, exposure to health promotion interventions, health facility level, and country.

**Results:**

Equipment, inputs, and staffing of facilities improved after the Initiative in both intervention and comparison areas. After adjustment for covariates, institutional delivery increased between baseline and follow-up by 3.1 percentage points (β = 0.031, 95% CI -0.03, 0.09) more in intervention areas than in comparison areas. The proportion of women in intervention areas who chose a facility other than their closest one to attend the delivery decreased between baseline and follow-up by 13 percentage points (β = − 0.130, 95% CI -0.23, − 0.03) more than in the comparison group.

**Conclusions:**

Results indicate that women in intervention areas of SMI are more likely to go to their closest facility to attend delivery after the Initiative has improved facilities’ capacity, suggesting that results-based aid initiatives targeting poor populations, like SMI, can increase the use of facilities closest to the place of residence for delivery care services. This should be considered in the design of interventions after the COVID-19 pandemic may have changed health and social conditions.

**Supplementary Information:**

The online version contains supplementary material available at 10.1186/s12884-021-04279-2.

## Background

Maternal and child health has improved in recent years, but health conditions related to pregnancy and delivery and in children under five are still an important contributor to the burden of disease in Mesoamerica [1, 2]. Institutional delivery, defined as a delivery attended in a health care institution, has been identified as an effective strategy to reduce maternal mortality and improve maternal health through emphasizing quality of care [3–7]. Based on one of the Sustainable Development Goals [8], low- and middle-income countries have implemented actions to increase the coverage and quality of institutional delivery.

The Salud Mesoamérica Initiative (SMI) is a public-private collaboration aimed to improve maternal and child health conditions in the poorest populations of Mesoamerica since 2011 through a comprehensive health care strategy to improve access to, use of, and quality of maternal, reproductive, neonatal, and pediatric health services. It is coordinated by the Inter-American Development Bank, and, through a results-based aid mechanism, works with countries in the region to reduce health disparities in geographic areas with the highest proportion of the population living in poverty [9].

Data on background conditions of coverage and quality of institutional delivery among poor populations in Mesoamerica were collected prior to the start of SMI as an input for the design of its interventions. The main barriers to access to institutional delivery include economic and geographic barriers to travel to health facilities, including roads and transportation networks and availability of public or private transportation; lack of proper infrastructure and human resources in health facilities; negative perceptions about the health services; and cultural and religious barriers [10–12].

This paper refers to three countries participating in SMI: Honduras, Nicaragua, and Guatemala. There are substantial differences in the coverage of institutional delivery across these countries, with very low coverage of institutional delivery in Guatemala (57.5%) as compared to Nicaragua (89.6%) or Honduras (74.0%) by 2017 [13]. Health services in Guatemala, Honduras, and Nicaragua are provided by different public and private providers [14–16]. Ministries of Health (MOHs) provide services free-of-cost, either through government-managed providers (Guatemala and Nicaragua) or by government-funded healthcare network managers (Honduras) to anyone who requests the services, and perform health promotion activities. In SMI target areas, MOHs are the main (and often the sole) healthcare service providers. Following national norms, routine delivery care is provided only in facilities with capacity to provide either basic or comprehensive essential obstetric and neonatal care (EONC). A substantial proportion of deliveries are attended by traditional birth attendants in Guatemala [17].

Previous work has documented barriers and facilitators for institutional delivery in these countries and found that women travel to more distant facilities to attend their deliveries, either complicated or uncomplicated, if they perceive facilities located farther away to be of better quality [18]. By improving the capacity of health facilities and with interventions to strengthen the demand for delivery care, it is expected that SMI can have a positive impact on delivery care and the selection of facilities to attend delivery among the population.

Therefore, we seek to evaluate if the Initiative has improved the capacity of health facilities to attend deliveries and if the patterns of delivery service utilization changed by assessing the impact of SMI on three outcomes: the availability of providers, equipment, and supplies, as well as quality of care around delivery (capacity of health facilities); the proportion of deliveries occurring in health facilities, identifying associated factors; and the proportion of women who attend a facility other than the closest one to give birth, identifying factors associated with this outcome.

## Methods

### Description of SMI

SMI was planned from the beginning to have two to three interconnected phases. A first phase of SMI (2013–2014) supported activities to improve the availability of staff, equipment, and supplies in MOH health facilities in all participating countries —such as improving supply chains and inventory management systems, designing and beginning implementation of the EONC strategy, reviewing staffing policies, and creating capacities for emergency obstetric and neonatal care. In a second phase (2015–2017), SMI incorporated supply-side and demand-side interventions aiming to improve the coverage and quality of care. In addition to previous interventions, interventions in this phase included the implementation of community and individual birth plans, behavior change communication strategies with cultural adaptations (specifically targeted media, speakers, messages, images, and languages), establishing or improving community platforms, strategies for early catchment of pregnant women in communities and facilities, funding systems for the transportation of pregnant women, operation of maternity homes, strengthening referral systems, establishing user management systems, training traditional birth attendants to detect risks and refer cases, implementing continuous quality improvement strategies and health care network management initiatives (for example, managerial electronic dashboards, local implementation plans, improved supervision tools, etc.), among others [19–21]. The third phase is currently in progress in Nicaragua and Honduras.

### Design

The SMI impact evaluation has a quasi-experimental design, with three partial-panel measurements in health facilities and two repeated cross-sectional measurements in households. This analysis uses information collected in health facilities and households in intervention and comparison areas of the Initiative at baseline (2011–2013) and following implementation of two phases of interventions (2017–2018).

### Population and sample

This analysis is restricted to Honduras, Nicaragua, and Guatemala, countries for which we have the required information for this analysis. Further details on SMI measurement methodology have been published elsewhere [22]. In summary, in each country, SMI administrators identified municipalities based on two criteria: first, those with the highest concentration of population in the poorest income quintiles according to national poverty estimates, and second, considering their proximity to enable interventions in the entire healthcare network. In Nicaragua, intervention municipalities were selected according to the concentration of the population in extreme poverty based on unmet basic needs; in Honduras, municipalities were selected according to the national poverty index and healthcare network manager (healthcare network managers with the most municipalities with high poverty were randomized in intervention and comparison groups); and in Guatemala, intervention municipalities were selected according to the concentration of extreme poverty, from departments with the largest concentration of the population in extreme poverty. Next, they identified municipalities with similar levels of poverty in Nicaragua and Guatemala to serve as comparison areas.

To get information from households, we conducted our own census in a set of communities selected with probability proportional to size to identify eligible households with women aged 15 to 49 years and children under 5. Among eligible households, we selected a random sample of 30 households per community to conduct the full SMI survey. To get information from health facilities, at the baseline, among the facilities that provide services to communities selected for the household survey, facilities that provide ambulatory EONC were selected at random from the MOH roster of facilities. Given the small number of facilities providing basic or comprehensive EONC in the study areas, we included in our sample all facilities of those levels in the intervention and comparison areas. We used a similar procedure for the selection of health facilities in the follow-up measurement, with the only difference that half of selected ambulatory-level facilities included in the follow-up sample were selected from facilities visited in the baseline measurement. Due to safety issues, data collection in Nicaragua during the baseline measurement had to be interrupted, allowing us to get information from 31% of the facilities providing basic or comprehensive EONC in the proposed sample.

### Data collection

The baseline surveys were conducted in Honduras between January 17 and June 1, in Nicaragua between March 1 and August 29, and in Guatemala between April 15 and August 11, all in 2013. Follow-up data were collected in Honduras between May 29 and October 25, 2017, in Nicaragua between June 14 and December 20, 2017, and in Guatemala between May 7 and August 29, 2018.

SMI used a computer-assisted personal interviewing (CAPI) system for data collection. The household survey was conducted at each household by trained interviewers, who read the questions to the interviewees and registered the answers in the computer, and assessed utilization of health services, barriers to care, and population health outcomes alongside health system infrastructure and delivery care components. Interviews with indigenous-language speakers were conducted in the corresponding indigenous languages by interviewers fluent in them, using a standard translation. Respondents were asked to indicate which health facilities were visited for different types of care, allowing us to link household information with facility conditions and services. The household survey included an interview of the head of household or person best informed about the household to collect information on household services and materials, ownership of assets (durable goods, land, livestock), household expenses, and sources of health care financing. All women of reproductive age (15 to 49 years) in the household were interviewed about their demographic characteristics, access to health care, current health status, recent history of illness and associated medical expenses, birth history, knowledge and use of family planning methods, exposure to health interventions, and satisfaction with community health workers. Women with children 0 to 59 months old were asked questions about health conditions and health services utilization of each child.

The health facility survey was conducted by trained medical personnel using CAPI and collected data on facility conditions, service provision and utilization, and quality of care. Health facilities were grouped according to three levels of EONC – ambulatory, basic, and comprehensive – following the official classification of each country. Mainly, ambulatory facilities provide outpatient care; basic facilities are able to attend uncomplicated vaginal deliveries and provide immediate emergency obstetric and neonatal care; and comprehensive facilities have a surgery room and are able to attend most obstetric and neonatal complications (not including intensive care). The facility director or person in charge of the health facility was interviewed by a trained interviewer in the facility to capture information on general facility characteristics, infrastructure, human resources, supply logistics, infection control, child health care, vaccine availability, family planning service provision, availability of contraceptives, and antenatal, delivery, and postpartum care services. Surveyors used an observation checklist to record direct observations of the availability and functionality, as applicable, of essential equipment and supplies required for maternal and child health care, including pharmaceuticals.

The health facility survey also included a medical record review (MRR) where information was extracted from a random sample of medical charts of women who had given birth in the facility in the 2 years prior to data collection. The methods for selection and review of records have been described elsewhere [22–24].

The study received institutional review board (IRB) approval from the leading institution and the MOH in each country. Methods were carried out in accordance with the national guidelines and regulations of participant countries and the Declaration of Helsinki. All women and personnel responsible for health facilities responding to the interviews signed informed consent forms prior to data collection. Identifying personal information was not collected in any component of the study.

### Study variables

#### Institutional delivery

**Fig. 1:**
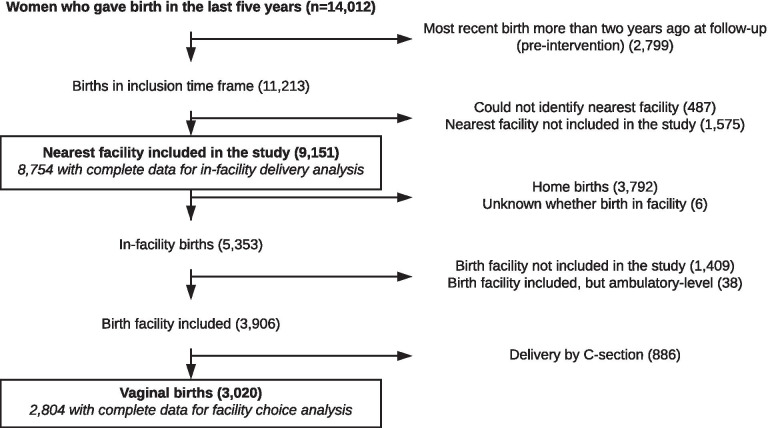
Sample composition

We define as institutional delivery any delivery that took place in a health facility regardless of facility type (public/private) or level (ambulatory, basic, or comprehensive EONC). For births in health facilities, our sample is restricted to births for which the nearest facility could be identified and was included in the study (see Fig. 1). The household survey collected information on the place of delivery for all births of women living in the household in the last 5 years. The analysis includes each woman’s most recent birth during the last 5 years in the baseline measurement. At the follow-up, only births from the last 2 years are included in the analysis in order to exclude births that took place before the SMI interventions were implemented.

#### Place of delivery and distance from home to delivery place

In addition to recording municipality and locality of residence, the household survey asked women about the name of the closest health facility, the health facility she usually attends, and the most recently visited health facility; the name and type of the facility she attended for her delivery (if any), and the self-reported distance (km), travel time (hours or minutes), and mode(s) of transport to the delivery facility. Because women typically reported ambulatory care facilities, which are limited to outpatient care and do not routinely provide delivery services, as their nearest and usual health facilities, we determine as the “closest” health facility for delivery a public establishment where the woman would be expected to deliver based on municipality and locality of residence, the referral network of the public health system, and a locality-by-locality review of the facilities women reported attending for delivery and for other health care needs. In this manner, we matched each selected community to its expected delivery facility. This matched facility is hereafter referred to as the “closest” facility for delivery.

The health system referral networks are based on municipal boundaries, and most municipalities have at least one basic or comprehensive EONC level facility, which in the majority of cases was also the nearest facility with capacity to attend delivery by travel time. Women in two municipalities (one in Nicaragua at the baseline, and one in Honduras in both rounds) matched to facilities that were not included in the health facility survey; we excluded these deliveries from the analysis of delivery location since facility-level variables were not measured at their closest facility.

#### Health facility characteristics and delivery capacity

We use the information from the health facility survey to construct a 6-point score of capacity to attend uncomplicated vaginal deliveries that included round-the-clock availability of skilled birth attendants (doctor or nurse, 2 points), availability of all basic equipment for antenatal and postpartum care (gynecological or exam table, lamp, obstetric tape measure, sphygmomanometer, and stethoscope, 1 point), and availability of basic inputs for delivery care such as oxytocin (to start labor, increase speed of labor, or stop bleeding after delivery, 1 point), methylergometrine or ergometrine maleate (to prevent excessive bleeding following childbirth, 1 point), and Ringer’s lactate/Hartmann’s solution or saline solution (for fluid resuscitation after blood loss, 1 point).

Additionally, we use information extracted from the charts of patients with uncomplicated vaginal deliveries at these health facilities to construct two indicators for quality delivery care as defined through SMI. First, the survey recorded whether the patient was administered oxytocin or another uterotonic after delivery according to standards for active management of the third stage of labor. Second, it collected information on immediate postpartum care, including checks of the mother’s temperature and blood pressure during the first 2 h after delivery and at discharge from the health facility.

#### Characteristics of delivery

The household questionnaire collected information on which health personnel were present during delivery, reasons for not delivering in a health facility (when the delivery occurred outside a health facility), accompaniment by a traditional birth attendant, type of delivery (planned C-section, emergency C-section, or vaginal delivery), seizures experienced during delivery, receipt of antenatal care, advice to have the delivery in a health facility, and advice to create a transportation plan.

#### Women and household characteristics

We collected information on the woman’s age, marital status, literacy, education (no school attendance, primary, secondary, high school, or university), occupation (housewife versus other [employed, student]), and number of previous pregnancies. In order to measure household conditions, we calculated a household asset index based on household assets (including piped water, improved toilets, and having a designated kitchen area, electricity, radio, stereo, television, telephone [mobile and fixed line], refrigerator, laundry machine, computer, bicycle, guitar, scooter, car, truck, land, cattle, mules, goats, chickens, or pigs). We collected information on the itemized monthly household expenditure as reported by the survey respondent and calculated per capita expenditure quintiles in each country and round. We use the countries’ census data [25–27] to define urban or rural status using 2500 inhabitants as a threshold.

### Statistical analysis

We analyze the impact of SMI on three outcome variables: institutional delivery (1 = delivery in a health unit vs. 0 = delivery outside a health unit); choosing a more distant facility for delivery (1 = delivery at a more distant facility vs. 0 = delivery at the closest facility) and the capacity score of facilities to attend deliveries. We restrict our sample to women who gave birth in a health facility, if the facility was included in the study, for the analysis of choice of delivery facility. Additionally, we exclude deliveries via C-section (to account only for uncomplicated deliveries) in the analysis of choice of delivery facility (see Fig. 1). We use a difference-in-difference approach fitting linear models to assess the impact of the intervention on the facility capacity score, institutional delivery, and delivering in a more distant facility. We include as covariates in our models the intervention or comparison group, measurement time (baseline or follow-up), and an interaction term between intervention and time, which is our impact estimate. In order to control for baseline differences between intervention and control groups, we adjust the models by baseline women’s characteristics (age, parity, education, household conditions, exposure to health interventions), health facility level, and country. In sensitivity analyses, we conduct the analysis substituting per capita itemized monthly household expenditure for asset indices in each model and find no meaningful changes in results. We fit models using Poisson and conditional logit specifications as robustness checks. Since results yield similar conclusions, only results from the linear models are presented.

We calculate the probability of women mentioning different reasons for not attending the delivery in a health institution from a series of logistic regression models. Since the choice of the place to attend delivery is not entirely an individual one, but it is influenced by other social and contextual factors, we group the reasons provided by women as cultural beliefs, finances and logistics, health facility limitations, and other, adjusted for age, education, parity, urban residence, asset index, and maternal literacy. Cultural beliefs include individual factors but also others related to the role of the family and community, like preferring labor under the care of a traditional birth attendant; preferring to give birth in the family home or another house; religious or cultural beliefs; wanting a traditional birth attendant to accompany; or being prevented from going by husband, partner, or another member of the family. Finances and logistics refers to reasons related to transportation problems, travel times, facility too distant, problems finding transportation, lacking someone to travel with, having no place to stay, not knowing where to go, and health facility charges for delivery. Health facility limitations included problems with health facilities (not having sufficient drugs or ill-equipped), problems with staff (not staffed, staff not trusted, not well informed or difficult to deal with, being previously treated poorly by the health facility), and having care denied when they have tried to go to a health facility. Other obstacles groups not being advised to deliver in a health facility and other reasons.

We used Stata version 15 [28] and R version 3 [29] for analyses. All analyses are conducted for the pooled sample and individually by country. Analyses using data from the household survey are weighted and adjusted for clustering and stratification in the sample design.

## Results

We collected information on 14,012 women with births in the last 5 years. Our analysis on institutional delivery is based on 8754 births in the inclusion time frame for which data for the nearest facility are included in the study. For the analysis on facility choice, after excluding births at facilities not surveyed during the study, births in ambulatory facilities, and home births, we end up with a sample of 3906 births that occurred in facilities in the study. Finally, excluding deliveries by C-section, 2804 births of unique women with complete data are available for inclusion in the analysis (Fig. 1).

Baseline characteristics of women included in the analysis are presented in Table 1, overall and for each country in intervention and comparison groups. Most of them live in rural areas (*n* = 3676, 64.1, 95% CI 49.1–76.7 for intervention group and *n* = 1804, 73.7, 95% CI 60.5–83.7 for the comparison group). Almost all women attended antenatal care before their deliveries (91.4% in intervention and 96.1% in comparison). However, a smaller proportion received counseling about facility delivery, were advised to give birth in a facility, and were informed they should have a C-section. Only 26.5% of women in the intervention area and 29.5% in comparison areas were advised to create a transportation plan. The exposure to these antenatal interventions also varies by country, being lower in Guatemala. Additional characteristics of women at baseline are presented in Additional file 1, Supplemental Table 1 and characteristics of women at follow-up are presented in Additional file 1, Supplemental Table 2.

**Table 1:** Baseline woman-level univariate characteristics of intervention and comparison groups, overall and by country

	All countries				Guatemala				Honduras				Nicaragua			
	Intervention		Comparison		Intervention		Comparison		Intervention		Comparison		Intervention		Comparison	
*Observations*	*3676*		*1804*		*2196*		*474*		*1119*		*771*		*361*		*559*	
	**%**	**95% CI**	**%**	**95% CI**	**%**	**95% CI**	**%**	**95% CI**	**%**	**95% CI**	**%**	**95% CI**	**%**	**95% CI**	**%**	**95% CI**
Age																
15–24	40.1	[36.9–43.4]	38.5	[35.3–41.9]	37.4	[35.2–39.7]	38.1	[32.7–43.9]	38.4	[35.9–41]	34.8	[30.1–39.8]	42.7	[36–49.6]	41.8	[36.8–46.9]
25–34	41.7	[38.9–44.5]	43.9	[40.8–47.1]	41.4	[38.7–44.1]	39.8	[35.7–44.1]	40.4	[37.1–43.8]	45	[39.3–50.8]	42.7	[37.2–48.5]	43.8	[39.6–48.1]
35–49	18.3	[16–20.8]	17.5	[15.2–20]	21.2	[19–23.6]	22.1	[16.7–28.5]	21.2	[18.3–24.4]	20.2	[16.6–24.4]	14.6	[10.7–19.6]	14.4	[11.3–18.3]
Primiparous	32.9	[29.8–36.1]	32.8	[29.8–36]	29.4	[27–31.9]	28.7	[21.1–37.8]	32.3	[29.1–35.7]	31.3	[28.3–34.5]	35.2	[29–42]	34.8	[29.2–40.9]
Area of residence																
Rural	64.1	[49.1–76.7]	73.7	[60.5–83.7]	85.6	[75.8–91.8]	69.6	[40.2–88.6]	90.7	[78.8–96.2]	84.1	[68–93]	34	[14.4–61.2]	65.5	[42–83.3]
Urban	35.9	[23.3–50.9]	26.3	[16.3–39.5]	14.4	[8.2–24.2]	30.4	[11.4–59.8]	9.3	[3.8–21.2]	15.9	[7–32]	66	[38.8–85.6]	34.5	[16.7–58]
Attended any antenatal care	91.4	[89.2–93.3]	96.1	[94.5–97.2]	78.3	[74.3–81.8]	90	[86.7–92.6]	95.1	[92.9–96.7]	97	[94.7–98.4]	96.2	[92.3–98.2]	96.3	[93.2–98.1]
Counseled regarding facility delivery	70	[64.7–74.8]	79.1	[75.4–82.4]	27.7	[24–31.6]	45.3	[39.1–51.6]	76.3	[71.2–80.7]	80.5	[74.2–85.5]	89.1	[84.6–92.5]	83.8	[78.1–88.2]
Advised to give birth in a facility	70.3	[64.7–75.2]	79.6	[75.8–82.9]	30.2	[26.2–34.6]	51.2	[43.1–59.3]	76.8	[71.5–81.5]	80.6	[74.4–85.5]	88	[81.5–92.4]	83.6	[77.3–88.4]
Informed should have a C-section	35.9	[29.6–42.7]	35.5	[31.3–39.8]	10.3	[8.2–12.9]	21.9	[15.7–29.6]	35	[29.5–40.9]	37.1	[30.6–44.1]	50.7	[39.9–61.4]	36.4	[30.1–43.2]
Advised to create a transportation plan	26.5	[23–30.4]	29.5	[24.5–35]	8.2	[6.5–10.4]	18.8	[14.8–23.7]	37.9	[32.2–44]	39.1	[30.6–48.5]	28.9	[22.5–36.4]	23.1	[16.9–30.7]

Overall, 33.3% of the facilities in intervention and 26.3% in comparison group were classified as comprehensive EONC level at baseline. Most of the facilities had a skilled birth attendant available (87.2 and 78.9% in intervention and comparison groups, respectively). The proportion of comprehensive-level facilities in the sample is lower in Guatemala and higher in Honduras and Nicaragua for both groups (Table 2).

**Table 2:** Facility-level univariate characteristics at baseline and follow-up for intervention and comparison groups, overall and by country

	Baseline, intervention		Follow-up, intervention		Baseline, comparison		Follow-up, comparison	
	%^a^	95% CI	%^a^	95% CI	%^a^	95% CI	%^a^	95% CI
**All countries**								
Mean facility score^b^	4.385	[3.99–4.78]	5.148***	[4.93–5.37]	4.105	[3.55–4.66]	4.880**	[4.41–5.35]
Comprehensive facility	33.3	[20.1–49.9]	22.2	[12.9–35.5]	26.3	[10.7–51.6]	36	[19.2–57.1]
Skilled birth attendant available	87.2	[72.1–94.7]	87	[74.9–93.8]	78.9	[53.5–92.4]	76	[54.6–89.3]
Doctors available 24 × 7	74.4	[58–85.9]	79.6	[66.5–88.5]	68.4	[43.4–85.9]	80	[58.8–91.8]
Nurses available 24 × 7	61.5	[45.1–75.7]	83.3**	[70.6–91.2]	47.4	[25.5–70.3]	64	[42.9–80.8]
*Gynecological/exam table observed*	84.6	[69.1–93.1]	96.3**	[86–99.1]	94.7	[67.5–99.4]	92	[71.5–98.1]
*Lamp observed/functioning*	82.1	[66.3–91.4]	98.1**	[87.5–99.8]	84.2	[58.7–95.2]	100**	–
*Obstetric/tape measure observed*	74.4	[58–85.9]	100***	–	78.9	[53.5–92.4]	100**	–
*Sphygmomanometer observed/functioning*	89.7	[75–96.2]	98.1*	[87.5–99.8]	89.5	[63.9–97.6]	92	[71.5–98.1]
*Stethoscope observed/functioning*	82.1	[66.3–91.4]	100**	–	89.5	[63.9–97.6]	96	[74.5–99.5]
All antenatal/postpartum care equipment	48.7	[33.2–64.5]	92.6***	[81.5–97.3]	52.6	[29.7–84]	7.3	[94.2–91.1]
Observations	39		54		19		25	
**Guatemala**								
Mean facility score^b^	4.353	[3.65–5.06]	5.556***	[5.25–5.86]	4.286	[3.59–4.99]	5.000*	[3.93–6.07]
Comprehensive facility	23.5	[8.4–50.8]	16.7	[5–43.2]	14.3	[1.2–70.1]	14.3	[1.2–70.1]
Skilled birth attendant available	88.2	[60.3–97.4]	94.4	[66–99.3]	85.7	[29.9–98.8]	85.7	[29.9–98.8]
Doctors available 24 × 7	70.6	[43.7–88.1]	72.2	[46.1–88.8]	71.4	[24.4–95.1]	100**	–
Nurses available 24 × 7	70.6	[43.7–88.1]	88.9*	[62.2–97.5]	71.4	[24.4–95.1]	71.4	[24.4–95.1]
*Gynecological/exam table observed*	82.4	[54.8–94.7]	88.9	[62.2–97.5]	85.7	[29.9–98.8]	71.4	[24.4–95.1]
*Lamp observed/functioning*	88.2	[60.3–97.4]	100*	–	71.4	[24.4–95.1]	100**	–
*Obstetric/tape measure observed*	82.4	[54.8–94.7]	100**	–	71.4	[24.4–95.1]	100**	–
*Sphygmomanometer observed/functioning*	82.4	[54.8–94.7]	100**	–	100	–	100	–
*Stethoscope observed/functioning*	82.4	[54.8–94.7]	100**	–	100	–	100	–
All antenatal/postpartum care equipment	47.1	[24.1–71.3]	88.9**	[62.2–97.5]	42.9	[10.4–82.9]	71.4	[24.4–95.1]
Observations	17		18		7		7	
**Honduras**								
Mean facility score^b^	4.571	[4.14–5.01]	5.071*	[4.38–5.77]	3.909	[2.99–4.83]	5.083**	[4.3–5.87]
Comprehensive facility	42.9	[18.9–70.7]	42.9	[18.9–70.7]	36.4	[12.4–69.8]	50	[21.9–78.1]
Skilled birth attendant available	92.9	[58–99.2]	64.3	[35–85.7]	72.7	[37.1–92.3]	66.7	[34.2–88.5]
Doctors available 24 × 7	85.7	[53.5–96.9]	57.1	[29.3–81.1]	63.6	[30.2–87.6]	66.7	[34.2–88.5]
Nurses available 24 × 7	42.9	[18.9–70.7]	57.1	[29.3–81.1]	27.3	[7.7–62.9]	50	[21.9–78.1]
*Gynecological/exam table observed*	92.9	–	100	–	100	–	100	–
*Lamp observed/functioning*	92.9	[58–99.2]	100	–	90.9	[49.1–99]	100	–
*Obstetric/tape measure observed*	78.6	[47.3–93.7]	100**	–	90.9	[49.1–99]	100	–
*Sphygmomanometer observed/functioning*	91.7	[52.5–99.1]	92.9	[58–99.2]	81.8	[44.1–96.3]	100*	–
*Stethoscope observed/functioning*	78.6	[47.3–93.7]	100**	–	81.8	[44.1–96.3]	100*	–
All antenatal/postpartum care equipment	64.3	[35–85.7]	92.9**	[58–99.2]	63.6	[30.2–87.6]	100**	–
Observations	14		14		11		12	
**Nicaragua**								
Mean facility score^b^	4.125	[2.75–5.5]	4.864	[4.66–5.07]	5.000	–	4.333	3.48–5.19
Comprehensive facility	37.5	[9.6–77.1]	13.6	[4.2–36.5]	0	–	33.3**	5.1–82.2
Skilled birth attendant available	75	[30.3–95.4]	95.5*	[71.4–99.4]	100	–	83.3	23–98.8
Doctors available 24 × 7	62.5	[22.9–90.4]	100**	–	100	–	83.3	23–98.8
Nurses available 24 × 7	75	[30.3–95.4]	95.5*	[71.4–99.4]	100	–	83.3	23–98.8
*Gynecological/exam table observed*	75	[30.3–95.4]	100*	–	100	–	100	–
*Lamp observed/functioning*	50	[15.8–84.2]	95.5**	[71.4–99.4]	100	–	100	–
*Obstetric/tape measure observed*	50	[15.8–84.2]	100**	–	0	–	100	–
*Sphygmomanometer observed/functioning*	87.5	[35.8–98.9]	100	–	100	–	66.7	17.8–94.9
*Stethoscope observed/functioning*	87.5	[35.8–98.9]	100	–	100	–	83.3	23–98.8
All antenatal/postpartum care equipment	25	[4.6–69.7]	95.5***	[71.4–99.4]	0	–	66.7***	17.8–94.9
Observations	8		22		1		6	

^a^Or mean, where specified

^b^Facility score is a 6-point score of the capacity to attend normal deliveries that included round-the-clock availability of skilled birth attendants, availability of basic equipment for antenatal and postpartum care (exam table, lamp, tape measure, sphygmomanometer, and stethoscope), and availability of basic inputs for delivery care such as oxytocin, methylergometrine or ergometrine maleate, and Ringer’s lactate/Hartmann’s solution or saline solution

* *p* < 0.1, ** *p* < 0.05, *** *p* < 0.01; two-sample, one-sided z-test of difference between baseline and follow-up estimate in each study group

### Impact of SMI on health facility delivery capacity

We first explore if SMI contributed to an increase in the capacity of health facilities for delivery care. Table 2 presents the mean facility capacity score, proportion of comprehensive facilities, availability of skilled birth attendant, and presence of doctors 24 × 7 and nurses 24 × 7 for intervention and comparison groups at baseline and follow-up measurements, as well as the availability of inputs for antenatal and postpartum care. When analyzing all countries together, the mean facility capacity score increased between baseline and follow-up in both intervention and comparison groups (4.39 to 5.15 in intervention group and 4.11 to 4.88 in comparison group). There were increases in the availability of doctors and nurses 24 × 7 in both groups, and in the availability of skilled birth attendants in the intervention group in Nicaragua and Guatemala only. We observe the same patterns in each country except Nicaragua, where we have a very reduced sample size for the baseline measurement.

We then analyze the impact of SMI on the capacity score, adjusting by level of facility and country (Additional file 1, Supplemental Table 3). After this adjustment, facilities had a score 0.78 points higher after the intervention than at baseline (95% CI 0.17, 1.39). Although positive, the effect of the intervention on the capacity score was not significantly different from zero (β = 0.25, 95% CI -0.32, 0.81). Facilities of comprehensive level had higher capacity than those of basic level, with an adjusted mean 0.65 points higher (95% CI 0.26, 1.04), and the capacity score was lower in Nicaragua as compared to Guatemala (β = − 0.523, 95% CI -0.97, − 0.08).

We also analyze the medical record data to assess changes in the delivery of care to national standards in the health facilities (Additional file 1, Supplemental Table 4). Immediate postpartum checks to standard (mother’s temperature and blood pressure are checked four times during the first hour after delivery and twice during the second hour, as well as at the time of discharge from the health facility) increased in intervention areas in Honduras (from 36.3 to 88.2%) and Guatemala (from 14.6 to 38%). Administration of uterotonics after delivery also improved in intervention areas of Guatemala from 79.8 to 98.4% and was measured to be above 90% in Nicaragua and Honduras.

### Impact of SMI on institutional delivery

**Fig. 2:**
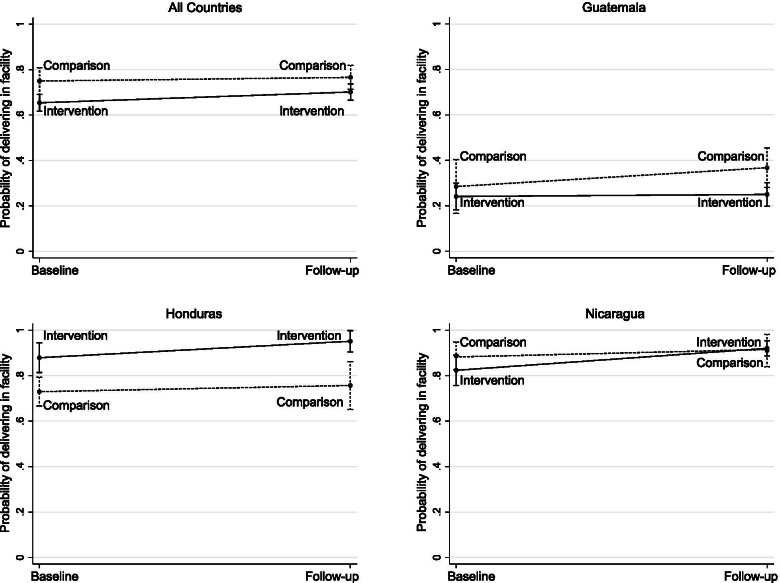
Probability of delivering in a health facility predicted from multivariate difference-in-difference models at baseline and follow-up in intervention and comparison groups, overall and by country

Overall, we observe no change in the proportion of institutional deliveries between baseline and follow-up in the intervention group (68.4 to 69.8%) and a decrease in the comparison group (82.6 to 63.1%) (Table 3). There are variations by country, with Guatemala having a notable increase in institutional deliveries in the intervention areas, and an even larger-magnitude decrease in comparison areas. In Honduras, there was a large increase in institutional delivery in both groups; no differences were found in Nicaragua. Multivariate analysis with a fixed effect on the woman’s closest facility (Table 4) finds that, after adjusting by women’s characteristics, health facility characteristics, and country, institutional delivery was 9.6 percentage points lower in intervention than comparison areas (β = − 0.096, 95% CI -0.18, − 0.01). However, SMI had a positive although not significantly different from zero impact of 3.1 percentage points on institutional delivery, as denoted by the interaction term of this model (β = 0.031, 95% CI -0.03, 0.09). Institutional delivery was higher in primiparous women (β = 0.074, 95% CI 0.05, 0.10), women in urban areas (β = 0.099, 95% CI 0.06, 0.14), and women with higher asset index and education. Institutional delivery was 21.6 percentage points higher in women with antenatal care (β = 0.216, 95% CI 0.17, 0.27) and among those who received counseling about health facility delivery and C-section. These patterns are present in all countries, although in Guatemala institutional delivery was 7.4 percentage points lower in the multilingual population (β = − 0.074, 95% CI -0.13, − 0.01) and 10 percentage points lower in women who speak indigenous languages only (β = − 0.10, 95% CI -0.17, − 0.03) as compared to those who only speak Spanish. Institutional delivery was 52.1 percentage points higher in Honduras (β = 0.521, 95% CI 0.45, 0.59) and 48.5 percentage points higher in Nicaragua (β = 0.485, 95% CI 0.40, 0.57) as compared to Guatemala. Adjusted effects of SMI on delivery attendance overall and for each country are presented in Fig. 2.

**Table 3:** Proportion of institutional deliveries and deliveries in a distant facility in intervention and control areas at baseline and follow-up, overall and by country

	Baseline, intervention			Follow-up, intervention			Baseline, comparison			Follow-up, comparison		
	N	%	95% CI	N	%	95% CI	N	%	95% CI	N	%	95% CI
**All countries**												
*Institutional delivery*	3676	68.4	[61.4–74.6]	2409	69.8	[66.2–73.1]	1804	82.6	[79–85.6]	865	63.1	[56.5–69.2]
*Delivery at a more distant facility*	953	26.9	[18.5–37.3]	879	29.2	[24.1–34.8]	683	30.8	[23.2–39.5]	289	39.4	[29.5–50.2]
**Guatemala**												
*Institutional delivery*	2196	22.6	[18.2–27.5]	874	29.2	[23.5–35.6]	474	39.3	[26.9–53.2]	345	29.5	[20.7–40.2]
*Delivery at a more distant facility*	286	44.6	[31.6–58.4]	142	36.8	[26.7–48.3]	83	52.9	[37.9–67.4]	47	54.2	[36.5–70.9]
**Honduras**												
*Institutional delivery*	1119	76	[67.3–83]	687	90.4	[85.6–93.7]	771	82.4	[76.4–87.2]	192	94.7	[83.2–98.5]
*Delivery at a more distant facility*	479	43.3	[35.1–52.0]	388	43.6	[34.7–52.8]	381	56.9	[45.3–67.8]	100	64.5	[43.1–81.4]
**Nicaragua**												
*Institutional delivery*	361	88.6	[77.5–94.6]	848	87	[82.3–90.6]	559	90.1	[84.6–93.9]	328	93.4	[87–96.8]
*Delivery at a more distant facility*	188	15.5	[6.5–32.3]	349	22.6	[16.2–30.6]	219	2.9	[0.7–10.8]	142	14.2	[6.8–27.3]

**Table 4:** Weighted OLS models predicting delivery in health facility^a^, overall and by country

	All		Guatemala		Honduras		Nicaragua	
	*β*	95% CI	*β*	95% CI	*β*	95% CI	*β*	95% CI
Round								
Baseline	0.000	Ref.	0.000	Ref.	0.000	Ref.	0.000	Ref.
Follow-up	0.016	[−0.03, 0.06]	0.083*	[− 0.01, 0.18]	0.028	[−0.06, 0.11]	0.023	[−0.02, 0.07]
Arm								
Comparison	0.000	Ref.	0.000	Ref.	0.000	Ref.	0.000	Ref.
Intervention	−0.096**	[− 0.18, − 0.01]	− 0.044	[− 0.21, 0.12]	0.150**	[0.03, 0.26]	−0.063	[− 0.18, 0.05]
Interaction term: Follow-up x Intervention	0.031	[−0.03, 0.09]	− 0.074	[− 0.19, 0.04]	0.045	[− 0.05, 0.14]	0.073	[− 0.03, 0.17]
Age								
15–24	0.000	Ref.	0.000	Ref.	0.000	Ref.	0.000	Ref.
25–34	0.016	[−0.01, 0.04]	0.036	[−0.01, 0.08]	0.018	[−0.01, 0.05]	0.009	[−0.02, 0.04]
35–49	0.014	[−0.02, 0.05]	0.045	[−0.01, 0.11]	0.006	[−0.05, 0.06]	0.013	[−0.03, 0.06]
Primiparous	0.074***	[0.05, 0.1]	0.128***	[0.07, 0.18]	0.072***	[0.04, 0.1]	0.050***	[0.02, 0.08]
Area of residence								
Rural	0.000	Ref.	0.000	Ref.	0.000	Ref.	0.000	Ref.
Urban	0.099***	[0.06, 0.14]	0.208***	[0.14, 0.28]	0.026	[−0.04, 0.1]	0.089***	[0.03, 0.14]
Asset index								
Low	0.000	Ref.	0.000	Ref.	0.000	Ref.	0.000	Ref.
Medium	0.043***	[0.02, 0.06]	0.034*	[0, 0.07]	0.060***	[0.02, 0.1]	0.028**	[0, 0.06]
High	0.045**	[0.01, 0.08]	0.052*	[−0.01, 0.11]	0.072**	[0.01, 0.13]	−0.003	[− 0.06, 0.06]
Languages spoken (Guatemala)								
Spanish only	–	–	0.000	Ref.	–	–	–	–
Spanish multilingual	–	–	−0.074**	[− 0.13, − 0.01]	–	–	–	–
Indigenous only	–	–	− 0.100***	[− 0.17, − 0.03]	–	–	–	–
Highest level of education attained								
None	0.000	Ref.	0.000	Ref.	0.000	Ref.	0.000	Ref.
Primary	0.012	[− 0.04, 0.06]	0.034	[−0.03, 0.1]	0.002	[−0.11, 0.11]	0.003	[−0.07, 0.07]
Secondary	0.053*	[0, 0.11]	0.156***	[0.06, 0.26]	−0.000	[−0.12, 0.12]	0.032	[−0.04, 0.11]
High school or higher	0.067**	[0, 0.13]	0.213***	[0.1, 0.33]	0.031	[−0.09, 0.15]	0.024	[−0.06, 0.11]
Literacy								
Cannot read at all	0.000	Ref.	0.000	Ref.	0.000	Ref.	0.000	Ref.
Able to read a portion	0.030	[−0.02, 0.08]	−0.005	[− 0.07, 0.06]	0.084	[− 0.02, 0.19]	0.003	[− 0.07, 0.08]
Able to read	0.110***	[0.06, 0.16]	0.061*	[−0.01, 0.13]	0.171***	[0.06, 0.28]	0.070**	[0, 0.14]
Marital status								
Single	0.000	Ref.	0.000	Ref.	0.000	Ref.	0.000	Ref.
Married	0.003	[−0.03, 0.03]	0.034	[− 0.04, 0.1]	0.013	[− 0.05, 0.08]	− 0.024	[− 0.06, 0.02]
Domestic partnership	0.000	[− 0.03, 0.03]	0.001	[− 0.07, 0.07]	0.047*	[− 0.01, 0.1]	− 0.016	[− 0.06, 0.03]
Other	−0.001	[− 0.05, 0.04]	0.026	[− 0.08, 0.14]	0.033	[− 0.07, 0.14]	− 0.032	[− 0.08, 0.02]
Housewife	0.019	[− 0.01, 0.05]	0.003	[− 0.07, 0.07]	− 0.018	[− 0.07, 0.03]	0.024*	[0, 0.05]
Owns a car or scooter	0.011	[−0.02, 0.04]	0.048	[− 0.02, 0.12]	0.033	[−0.01, 0.07]	− 0.002	[− 0.04, 0.03]
Any ANC?	0.216***	[0.17, 0.27]	0.058**	[0.01, 0.1]	0.290***	[0.17, 0.41]	0.482***	[0.37, 0.59]
Counseled regarding health facility delivery	0.040***	[0.01, 0.07]	0.034	[− 0.02, 0.09]	− 0.003	[− 0.05, 0.05]	0.016	[− 0.03, 0.06]
Advised to give birth in a health facility	0.047***	[0.02, 0.08]	0.053**	[0.01, 0.1]	−0.011	[− 0.06, 0.04]	0.032	[−0.02, 0.08]
Informed should have a C-section	0.070***	[0.05, 0.09]	0.137***	[0.07, 0.2]	0.059***	[0.03, 0.09]	0.051***	[0.03, 0.07]
Advised to create a transportation plan	0.004	[−0.02, 0.03]	− 0.011	[− 0.08, 0.05]	0.018	[− 0.01, 0.05]	− 0.010	[− 0.03, 0.01]
Nearest facility type								
Basic	0.000	Ref.	0.000	Ref.	0.000	Ref.	0.000	Ref.
Comprehensive	0.098	[−0.22, 0.42]	0.014	[−0.24, 0.27]	0.162***	[0.06, 0.26]	−0.011	[− 0.06, 0.04]
Nearest facility score	−0.002	[− 0.02, 0.02]	0.050***	[0.02, 0.08]	0.006	[−0.02, 0.03]	−0.041*	[− 0.09, 0]
Provide facilities for relatives^b^	0.017	[−0.05, 0.09]	−0.188***	[− 0.29, − 0.09]	0.068	[− 0.03, 0.17]	0.019	[− 0.04, 0.07]
Country								
Guatemala	0.000	Ref.	–	–	–	–	–	–
Honduras	0.521***	[0.45, 0.59]	–	–	–	–	–	–
Nicaragua	0.485***	[0.4, 0.57]	–	–	–	–	–	–
***Observations***	*8754*		*3889*		*2769*		*2096*	

* *p* < 0.1, ** *p* < 0.05, *** *p* < 0.01

^a^With fixed effects at the facility level

^b^Includes accommodation or meals for relatives during hospital stay

We analyze the reasons given by women for not delivering in a facility for each country. In Guatemala, the most mentioned reasons for not delivering in a facility were related to culture, family, and beliefs, both in intervention groups (60.9% at baseline and 80.3% at follow-up) and in comparison groups (49% at baseline and 86.1% at follow-up) (Additional file 1, Supplemental Table 5). In Honduras, women reported reasons related to finances and logistics in intervention (64.4% at baseline and 73.2% at follow-up) and comparison groups (52.7% at baseline and 38.4% at follow-up). Finally, in Nicaragua, the main reasons for not delivering in a facility were related to culture, family, and beliefs in the intervention group (59.2% at baseline and 35.6% at follow-up), while women in the comparison group mostly mentioned other reasons (like not being advised to deliver in a health facility), with 45.1% in intervention and 30.8% in comparison group.

### Impact of SMI on choice of a more distant facility

The unadjusted analysis among all countries shows a small increase in the proportion of women who gave birth in a facility other than the closest one between baseline and follow-up in the intervention group, though this pooled statistic conceals a large-magnitude downward trend in Guatemala (from 44.6 to 36.8%) and an upward one in Nicaragua (from 15.5 to 22.6%) (Table 3). In the comparison group, the proportion of women who delivered in a distant facility stayed constant in Guatemala and increased notably in Honduras and in Nicaragua (where more women chose the closest facility for delivery).

**Fig. 3:**
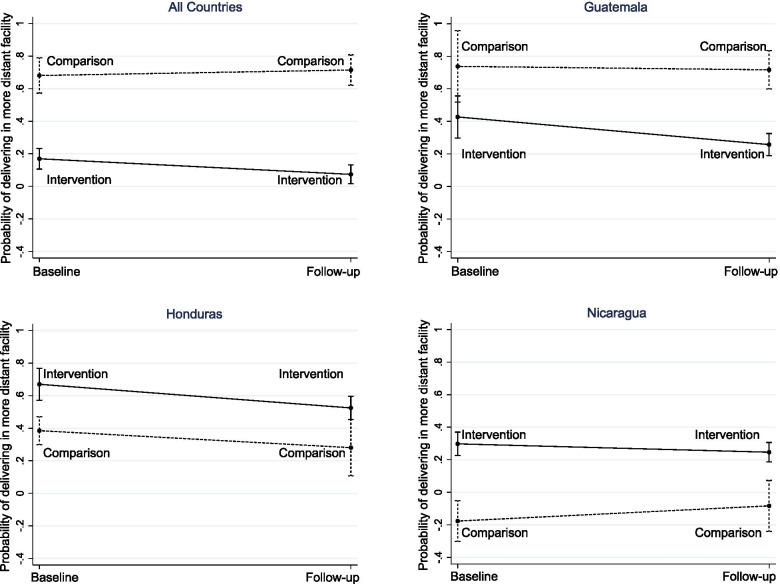
Probability of choosing a more distant facility for delivery predicted from multivariate difference-in-difference models at baseline and follow-up in intervention and comparison groups, overall and by country

However, when we control for women’s characteristics, health facility characteristics, and country in a multivariate model (Table 5), we find that women in intervention areas were 51.2 percentage points less likely to deliver in a more distant facility than in comparison areas (β = − 0.512, 95% CI -0.67, − 0.35), and find a positive impact of SMI, with a reduction between baseline and follow-up of 13 percentage points (β = − 0.130, 95% CI -0.23, − 0.03) in the intervention group as compared to the comparison group. Women were more likely to deliver in a more distant facility when the facility attending the delivery had a capacity score higher than that of the closest facility (β = 0.183, 95% CI 0.13, 0.24). Primiparous women, women who attended school, women who attended antenatal care, and women who had emergency deliveries were more likely to give birth in a more distant facility. These patterns remain in all countries. In contrast to the unadjusted results, women in Nicaragua were 39.4 percentage points more likely to choose a distant facility for delivery than women in Guatemala (β = 0.394, 95% CI 0.21, 0.58). Adjusted effects of SMI on delivery attendance overall and for each country are presented in Fig. 3.

**Table 5:** Weighted OLS models predicting delivery in a more distant facility^a^, overall and by country

	All		Guatemala		Honduras		Nicaragua	
	*β*	95% CI	*β*	95% CI	*β*	95% CI	*β*	95% CI
Round								
Baseline	0.000	Ref.	0.000	Ref.	0.000	Ref.	0.474***	Ref.
Follow-up	0.034	[−0.05, 0.11]	− 0.033	[− 0.21, 0.17]	0.017	[− 0.27, 0.06]	0.093*	[− 0.01, 0.2]
Arm								
Comparison	0.000	Ref.	0.000	Ref.	0.000	Ref.	0.000	Ref.
Intervention	−0.512***	[− 0.67, − 0.35]	−0.311**	[− 0.61, − 0.01]	0.285***	[0.12, 0.45]	0.474***	[0.29, 0.66]
Follow-up x Intervention	−0.130**	[− 0.23, − 0.03]	−0.149	[− 0.39, 0.1]	−0.041	[− 0.22, 0.13]	−0.145**	[− 0.28, − 0.01]
Age								
15–24	0.000	Ref.	0.000	Ref.	0.000	Ref.	0.000	Ref.
25–34	− 0.014	[− 0.05, 0.02]	− 0.023	[− 0.11, 0.06]	0.005	[− 0.04, 0.06]	−0.019	[− 0.08, 0.04]
35–49	0.012	[−0.04, 0.07]	− 0.068	[− 0.17, 0.03]	0.079	[− 0.01, 0.15]	−0.025	[− 0.11, 0.06]
Primiparous	0.060***	[0.02, 0.1]	0.017	[−0.06, 0.1]	0.103***	[0.03, 0.15]	0.043	[−0.02, 0.1]
Area of residence								
Rural	0.000	Ref.	0.000	Ref.	0.000	Ref.	0.000	Ref.
Urban	−0.020	[− 0.07, 0.03]	0.084*	[− 0.01, 0.18]	− 0.021	[− 0.14, 0.04]	−0.031	[− 0.09, 0.03]
Asset index								
Low	0.000	Ref.	0.000	Ref.	0.000	Ref.	0.000	Ref.
Medium	−0.004	[− 0.04, 0.03]	− 0.005	[− 0.09, 0.08]	0.055**	[0, 0.11]	− 0.031	[− 0.07, 0.01]
High	0.005	[− 0.05, 0.06]	− 0.013	[− 0.12, 0.09]	0.033	[− 0.04, 0.1]	− 0.011	[− 0.1, 0.08]
Languages spoken (Guatemala)								
Spanish only	–	–	0.000	Ref.	–	–	–	–
Spanish multilingual	–	–	−0.077*	[− 0.16, 0.01]	–	–	–	–
Indigenous only	–	–	0.055	[−0.2, 0.11]	–	–	–	–
Has attended school	0.026***	[0.01, 0.04]	0.080***	[0.03, 0.11]	0.046**	[0.01, 0.08]	0.006	[−0.02, 0.03]
Literacy								
Cannot read at all	0.000	Ref.	0.000	Ref.	0.000	Ref.	0.000	Ref.
Able to read a portion	−0.019	[− 0.09, 0.05]	− 0.025	[− 0.15, 0.1]	0.069	[− 0.02, 0.16]	− 0.071	[− 0.17, 0.03]
Able to read	− 0.042	[− 0.11, 0.03]	−0.041	[− 0.2, 0.12]	0.020	[− 0.06, 0.1]	−0.081*	[− 0.17, 0.01]
Marital status								
Single	0.000	Ref.	0.000	Ref.	0.000	Ref.	0.000	Ref.
Married	0.042*	[0, 0.09]	0.094*	[−0.01, 0.2]	0.001	[−0.08, 0.08]	0.043	[−0.01, 0.1]
Domestic partnership	0.006	[−0.04, 0.05]	0.074	[−0.02, 0.17]	− 0.013	[− 0.08, 0.05]	0.001	[− 0.05, 0.06]
Other	−0.006	[− 0.07, 0.06]	0.257***	[0.13, 0.38]	−0.022	[− 0.16, 0.12]	− 0.035	[− 0.11, 0.04]
Housewife	− 0.047**	[− 0.09, − 0.01]	0.057	[− 0.05, 0.17]	− 0.050	[− 0.13, 0.03]	− 0.061**	[− 0.11, − 0.01]
Owns a car or scooter	0.010	[− 0.03, 0.05]	− 0.028	[− 0.11, 0.05]	0.022	[− 0.06, 0.11]	0.018	[− 0.03, 0.07]
Type of visit								
Planned delivery	0.000	Ref.	0.000	Ref.	0.000	Ref.	0.000	Ref.
Emergency delivery	0.020	[−0.01, 0.05]	0.061	[−0.01, 0.13]	0.037	[−0.01, 0.09]	0.004	[− 0.04, 0.05]
Attended any antenatal care	0.033	[− 0.05, 0.12]	0.042	[−0.1, 0.18]	− 0.092	[− 0.26, 0.07]	0.061	[− 0.05, 0.17]
Counseled regarding facility delivery	−0.058*	[− 0.12, 0]	0.002	[− 0.11, 0.11]	− 0.036	[− 0.11, 0.04]	−0.047	[− 0.14, 0.05]
Advised to give birth in a facility	0.026	[−0.03, 0.08]	0.073	[−0.03, 0.18]	− 0.006	[− 0.09, 0.08]	0.014	[− 0.06, 0.09]
Informed should have a C-section	0.051**	[0.01, 0.1]	0.094*	[− 0.01, 0.19]	0.042	[−0.01, 0.1]	0.050	[− 0.02, 0.12]
Advised to create a transportation plan	0.021	[− 0.01, 0.06]	0.020	[− 0.05, 0.09]	−0.004	[− 0.05, 0.05]	0.044*	[0, 0.09]
Nearest facility type								
Basic	0.000	Ref.	0.000	Ref.	0.000	Ref.	0.000	Ref.
Comprehensive	−0.024	[− 0.15, 0.11]	− 0.100	[− 0.34, 0.14]	−0.133	[− 0.31, 0.05]	−0.036	[− 0.12, 0.05]
Facility capacity score difference^b^	0.183***	[0.13, 0.24]	0.150***	[0.09, 0.21]	0.163***	[0.11, 0.21]	0.236**	[0.05, 0.42]
Travel time to delivery facility								
< =30 min	0.000	Ref.	0.000	Ref.	0.000	Ref.	0.000	Ref.
31–60 min	0.095***	[0.05, 0.14]	0.421***	[0.32, 0.52]	0.147***	[0.06, 0.24]	0.020	[−0.03, 0.07]
1–2 h	0.223***	[0.16, 0.29]	0.552***	[0.45, 0.65]	0.332***	[0.23, 0.43]	0.091*	[0, 0.18]
> 2 h	0.346***	[0.27, 0.42]	0.706***	[0.55, 0.87]	0.500***	[0.41, 0.59]	0.172***	[0.07, 0.28]
Provides hospitalization services	0.119	[−0.07, 0.3]	0.026	[−0.22, 0.27]	–	–	0.004	[−0.26, 0.27]
Provides facilities for relatives^c^	−0.060	[− 0.18, 0.06]	0.113	[− 0.08, 0.3]	0.063	[− 0.08, 0.21]	− 0.022	[− 0.31, 0.27]
Country								
Guatemala	0.000	Ref.	–	–	–	–	–	–
Honduras	−0.294**	[−0.53, − 0.06]	–	–	–	–	–	–
Nicaragua	0.394***	[0.21, 0.58]	–	–	–	–	–	–
*Observations*	*2804*		*558*		*1348*		*898*	

* *p* < 0.1, ** *p* < 0.05, *** *p* < 0.01

^a^With fixed effects at the facility level

^b^score where delivery was attended minus score of the closest facility

^c^Includes accommodation or meals for relatives during hospital stay

Because complications experienced during delivery can be a motive to transfer a patient to a facility providing a superior level of EONC, we looked for evidence of complications during deliveries included in the survey. We found no evidence that complications were associated with delivery at a comprehensive EONC-level facility. Women who reported delivering in a comprehensive-level facility were about as likely to report bleeding (12.4, 95% CI 8.8–17.2) or seizures (3.7, 95% CI 2.6–5.3) as women who delivered at a basic-level facility (16.2, 95% CI 12.7–20.6, reported bleeding, and 3.7, 95% CI 2.7–5.1, reported seizures).

We examine whether women who had stayed in a maternity home were more likely to have delivered in a more distant health facility. However, we find evidence of the opposite pattern – in Honduras in intervention areas at follow-up, women who had stayed in a maternity home were substantially less likely to choose a distant facility (21.3, 95% CI 13.7–31.7; versus 53.7, 95% CI 40.49–66.4 of women who did not stay at a maternity home). In comparison areas, women chose to deliver at a more distant facility at the same rate regardless of use of a maternity home (67.2, 95% CI 44.8–83.8 of women using a maternity home; and 65.2, 95% CI 37.2–85.6 of women not using a maternity home). In Nicaragua, women were slightly more likely to choose a more distant facility for delivery if they did not use a maternity home (56.1, 95% CI 51.2–60.8, versus women who did stay at a maternity home, 49.46, 95% CI 42.9–56.0).

## Discussion

This is the first study documenting a decrease in the choice to travel farther to attend a delivery after SMI, an initiative aimed to improve access and quality of delivery care in Guatemala, Honduras and Nicaragua. We found that women were more likely to deliver in a more distant facility when the facility attending the delivery had a higher capacity than the closest facility, as measured by our capacity score. This is a crucial finding since attending deliveries at local facilities indicates the use of resources was optimized, which can lead to more efficient performance of the health system and improvements in health conditions. Although other factors could play a role, results suggest a shift in care-seeking behavior that could be related to changes in the supply of care, including increased capacity of a facility to attend deliveries and reorganization of the health care network. Findings from SMI could be used to improve health care in other regions by improving the supplies and practice at the local level.

It has been recognized that increasing access to health services without an improvement of quality of care is not enough to achieve health gains [30, 31]. In the countries under study, SMI has created competencies in human resources in the management of obstetric complications and strengthened referral systems in the intervention areas. SMI has been working to improve health conditions of the poorest population in Mesoamerica, generating great participation from countries and achieving positive results in healthcare coverage and quality related to maternal health, for example, an increase in the proportion of pregnancies with antenatal care, the proportion of maternal and neonatal complications attended according to the norms, and improvements in equipment and staffing of health facilities [9]. Although our study finds no significant impact of the intervention on the capacity of facilities to provide delivery care as measured by our capacity score, we find an improvement in specific aspects of equipment and staffing in intervention facilities, also documented in previous studies [32], which suggests that SMI has helped countries to improve the capacity of their health facilities. Most important, institutional delivery, and especially the choice of the closest facility for delivery care, was higher for facilities that had a higher capacity score, indicating that women are more likely to attend facilities that have the necessary equipment, supplies, and staff available. Given that women who attend public health facilities have little or no choice to select the personnel attending their delivery, choosing the right health facility matters.

The selection of place to attend a delivery is also affected by personal characteristics, some of which we were able to explore in this study. We found a higher proportion of institutional deliveries among women with more education and higher socioeconomic status. Institutional delivery was lower among multilingual women in Guatemala, the country with the highest proportion of multilingual population, which can also reflect differences in socioeconomic status of these groups. While SMI had a modest impact on institutional delivery itself, the analysis of the reasons for not delivering in a health facility indicates that cultural reasons are an important driver for choosing a home delivery, both before and after the intervention and in both intervention and comparison groups. Creating health services that are culturally sensitive and adapted to population needs should be a priority to encourage in-facility deliveries [33]. Because perceptions and beliefs about the capacity of health facilities can affect care-seeking for delivery services, the need to reduce cultural barriers that affect the utilization of delivery services may persist, as has been suggested by other studies in the region [34]. Furthermore, the decision to give birth in a health facility is not necessarily individualized: the woman’s partner, family and others in her community may also play a role [35].

Each country in this analysis has a public health system, organized as a network in which each facility is assigned a service population following geographical criteria, with a referral system in place where cases can transit from lower to more specialized levels of care, although referrals may face problems due to limited resources for transportation or functioning of facilities. Even when ambulatory healthcare facilities refer pregnant women for delivery care to a specific facility, which is usually the closest, women may decide to attend a different one. The selection of a place to receive delivery care is a complex process that involves individual aspects, but also cultural and social determinants (like race or socioeconomic status), other determinants of quality of care at the facility level and trust in health services. Under these conditions, SMI had a significant impact on the selection of facilities where delivery was attended, decreasing the likelihood that a woman chose to travel beyond her nearest birth facility to deliver. Attending delivery at the closest facility may reduce travel times for women, encourage institutional delivery, and reduce risks associated with delayed arrival to the health facility. In addition, delivery at the closest facility reduces the pressure on some facilities in the health systems of these countries, optimizing the capacity of the existing network. Although selecting a more distant facility for delivery is not necessarily harmful as long as women attend a facility with the proper capacity to attend a delivery, results of this study suggest that the improvement of facilities’ capacity to attend delivery supported by SMI has led to a reduction in “bypassing” behavior in cases in which the closest facility has adequate capacity, promoting a better use of health system resources.

The increased use of nearby facilities for delivery care is an important finding as we move toward universal health coverage (UHC). UHC implies financial protection but also the need for improved health system performance, so health services need to achieve a given level of quality to be effective [36]. Success in achieving UHC depends on the ability of health facilities to meet the health needs of the population. Although in our study we did not have a comprehensive measure of the level of quality of care, the index we used gives an approximation to the capacity of a facility to provide care with a minimum standard, which can be considered in the selection of a facility for delivery. The results of this study are encouraging for other countries as well, indicating that investing in health care, including not only inputs and equipment but also improvements to increase quality of care overall, and locating high-quality services close to communities, as SMI has done, can reduce costs for the health system and the population, and can encourage people to access services. This is especially important in countries like Nicaragua which have made substantial efforts to expand their health services network.

Furthermore, SMI intervention focused on the health sector, specifically the MOH, while broader political, economic, or societal changes would be necessary to address the social determinants of health. Our results show that health interventions can have an impact on health behaviors, but broader multisector interventions may be needed to achieve deeper and long-lasting impact. Other factors related to social conditions that may affect the decision-making process to select a facility for care play an important role as discrimination has been documented in these countries, especially in Guatemala with indigenous populations [37], and its role should not be denied. Although SMI aimed to improve the cultural competencies of health care providers, these interventions may be insufficient to fully address issues such as institutional racism, cultural racism and discrimination [38].

### Limitations

We recognize that the strongest evaluation model to assess the impact of an intervention like SMI would be an experimental design, in which areas are allocated at random to intervention and comparison groups, with pre and post measurements. A controlled experiment cannot be implemented on a large-scale health program. SMI instead has a quasi-experimental design in which areas with the highest concentration of poverty were assigned to receive the intervention, and comparison areas were identified in each country matching on socioeconomic characteristics. Because of this limitation, comparison and intervention groups are not always similar at baseline in observable and non-observable characteristics, including safety concerns and socioeconomic conditions. In addition, because the household measurement is not designed as a panel and the observation unit of the analysis is a birth, we do not have repeat measurements of the same women over time, but rather two independent cross-sectional samples of births of women in the same study area. To address these limitations, we employ a difference-in-difference approach, and adjust for a set of covariates to control for the effect of socio-demographic characteristics that may confound the estimation of the impact of SMI.

The information about health facility characteristics relies on observation and therefore is not prone to reporting bias. We faced limitations in the measurement of capacity of health care facilities. Our facility capacity index may have a limited ability to capture changes in the staffing and availability of inputs and equipment in the facility over time, and could not measure other aspects like competency of health professionals, waiting times, and availability of medications in a homogenous way across countries. We must recognize that having the minimum requirements of inputs and equipment does not fully explain quality of care. Due to small subsample sizes, it was not possible to incorporate quality of care indicators from the MRR to the capacity index. To overcome this limitation, we document the changes in staffing and specific equipment and inputs that may reflect progress in this area over time. The capacity score we constructed for this study builds on one we previously used that was able to distinguish levels of capacity in a sample of facilities included in the baseline measurement of SMI [18]. Further research is needed to understand the full effect of quality of care on the choice of place for delivery care.

Information from the surveys allows us to link the locality of residence of women with their closest facility (according to the service referral network) and the facility they actually attended for delivery. For confidentiality issues, we did not collect geographical coordinates of the households of participants. The matching method chosen may be imprecise in a few cases where women live far from the center of the locality, and may be inaccurate in the case that a woman was living or visiting outside the municipality of current residence at the time of delivery. In addition, our sample for the choice of health facility analysis is restricted to births for which the nearest facility could be identified and was included in the study. Given that we restrict our sample for the choice of health facility analysis, our results do not necessarily depict SMI’s impact on institutional delivery coverage.

Although the activities of SMI were focused in the areas selected for the intervention, it is possible they have a spillover effect, also generating changes in the health services in comparison areas. MOHs could scale-up SMI interventions to other areas of the country including comparison municipalities, which could have mitigated the impact of our evaluation. We anticipate higher potential for spillover effects in Nicaragua, where national policies encourage the application of innovations nationwide. In addition, due to the location of some comparison areas contiguous to intervention areas, it is possible for women in comparison areas to be referred or seek care at hospitals that also provide services to intervention areas. We would expect these biases to result in improved performance in comparison areas, in which case the results presented here may underestimate the true impact of the Initiative in intervention areas.

This study is based on large probabilistic samples of households and health facilities in the participating countries, and it was possible to link information from both sources as part of the analysis. However, the level of confidence to make inferences for individual countries is limited by sample size, especially in the case of Nicaragua. The three countries included in this analysis have very different coverage of institutional delivery, which is higher in Nicaragua and Honduras and lower in Guatemala. Although the sample size for each country limits our capacity to make reliable conclusions for each individually, the findings from pooled analysis across countries indicate that an improvement in the capacity of health facilities through an intervention like SMI can generate higher utilization by the population. The timing of the evaluation and the occurrence of the COVID-19 pandemic indicate we need to reflect about our results considering the changes the pandemic has brought in health systems.

## Conclusions

The results of this study indicate that SMI had a positive impact on delivery care in Nicaragua, Honduras, and Guatemala, reducing women’s election to travel to a more distant facility to give birth in favor of local facilities where the Initiative aimed to increase capacity and quality of delivery care. This study occurred before the COVID-19 pandemic, which may have set back many of the improvements achieved. The SMI final evaluation will account for the impact of COVID-19 at the household and facility level to make recommendations on how best to address the challenges posed by the pandemic. So far, the main lesson of this experience is that if we can improve delivery care with adequate supplies and actions to improve its quality, the population will attend health services closer to their place of residence instead of traveling farther to seek care, increasing convenience and reducing costs for individuals, and improving efficiency in the health system. This intervention model can be applied in other countries with similar social and health conditions.

## Supplementary Information

**Additional file 1: Supplemental Table 1.** Additional characteristics of women at baseline in intervention and comparison groups, overall and by country. **Supplemental Table 2.** Characteristics of women at follow-up in intervention and comparison groups, overall and by country. **Supplemental Table 3.** Unweighted OLS models predicting facility score, overall and by country. **Supplemental Table 4.** Results of medical record review of uncomplicated deliveries in the last 2 years at baseline and follow-up for intervention and comparison groups by country. **Supplemental Table 5.** Reasons for not delivering in facility at baseline and follow-up in intervention and comparison groups by country.

## Data Availability

The data underlying this article can be found in the following links for the baseline period: Honduras household: http://ghdx.healthdata.org/record/ihme-data/honduras-salud-mesoam%C3%A9rica-initiative-baseline-census-and-household-survey-2013 Honduras health facility: http://ghdx.healthdata.org/record/ihme-data/honduras-salud-mesoam%C3%A9rica-initiative-baseline-health-facility-survey-2013 Guatemala household: http://ghdx.healthdata.org/record/ihme-data/guatemala-salud-mesoam%C3%A9rica-initiative-baseline-census-and-household-survey-2013 Guatemala health facility: http://ghdx.healthdata.org/record/ihme-data/guatemala-salud-mesoam%C3%A9rica-initiative-baseline-health-facility-survey-2013 Nicaragua household: http://ghdx.healthdata.org/record/ihme-data/nicaragua-salud-mesoam%C3%A9rica-initiative-baseline-census-and-household-survey-2013 Nicaragua health facility: http://ghdx.healthdata.org/record/ihme-data/nicaragua-salud-mesoam%C3%A9rica-initiative-baseline-health-facility-survey-2013 Data from the follow-up period have not yet been published and will be shared on reasonable request to the corresponding author.

## Abbreviations

SMI: Salud Mesoamérica Initiative
MOH: Ministry of Health
EONC: Essential Obstetric and Neonatal Care
CAPI: Computer-Assisted Personal Interview
MRR: Medical record review
IRB: Institutional Review Board
