# Supplementary material for: Impact of the Salud Mesoamerica Initiative on delivery care choices in Guatemala, Honduras, and Nicaragua
Source: BMC Pregnancy Childbirth. 2022 Jan 3;22:5. doi: 10.1186/s12884-021-04279-2 (PMC8720941; doi:10.1186/s12884-021-04279-2)
Supplement: Supplementary file 1 — Additional file 1: Supplemental Table 1. Additional characteristics of women at baseline in intervention and comparison groups, overall and by country. Supplemental Table 2. Characteristics of women at follow-up in intervention and comparison groups, overall and by country. Supplemental Table 3. Unweighted OLS models predicting facility score, overall and by country. Supplemental Table 4. Results of medical record review of uncomplicated deliveries in the last 2 years at baseline and follow-up for intervention and comparison groups by country. Supplemental Table 5. Reasons for not delivering in facility at baseline and follow-up in intervention and comparison groups by country. [file 12884_2021_4279_MOESM1_ESM.docx]

**Additional file 1.**

**Supplemental Table 1. Additional characteristics of women at baseline in intervention and comparison groups, overall and by country**

|  | **All countries** | | | | **Guatemala** | | | | **Honduras** | | | | **Nicaragua** | | | |
| --- | --- | --- | --- | --- | --- | --- | --- | --- | --- | --- | --- | --- | --- | --- | --- | --- |
|  | **Intervention** | | **Comparison** | | **Intervention** | | **Comparison** | | **Intervention** | | **Comparison** | | **Intervention** | | **Comparison** | |
| *Observations* | *3676* |  | *1804* |  | *2196* |  | *474* |  | *1119* |  | *771* |  | *361* |  | *559* |  |
|  | **%** | **95% CI** | **%** | **95% CI** | **%** | **95% CI** | **%** | **95% CI** | **%** | **95% CI** | **%** | **95% CI** | **%** | **95% CI** | **%** | **95% CI** |
| Asset index |  |  |  |  |  |  |  |  |  |  |  |  |  |  |  |  |
| Low | 49.4 | [45-53.9] | 42.6 | [36.7-48.8] | 57.5 | [51.9-62.9] | 42 | [32.6-52.1] | 52.2 | [46.3-58.1] | 46.6 | [39.5-54] | 43 | [35.4-51] | 39.3 | [29-50.7] |
| Medium | 37.5 | [33.1-42.1] | 39.1 | [35-43.5] | 23.7 | [21.2-26.5] | 29.5 | [25.1-34.4] | 31.2 | [27.4-35.4] | 35.2 | [29.7-41.1] | 49.4 | [42.5-56.2] | 44.1 | [37-51.5] |
| High | 13.1 | [10.9-15.7] | 18.3 | [15.1-21.9] | 18.8 | [15.1-23] | 28.5 | [21.3-36.9] | 16.5 | [13.3-20.4] | 18.2 | [13.9-23.3] | 7.6 | [4.9-11.5] | 16.6 | [11.8-22.8] |
| Languages spoken (Guatemala) | |  |  |  |  |  |  |  |  |  |  |  |  |  |  |  |
| Spanish only | - | - | - | - | 20.5 | [14.7-27.9] | 54.9 | [33.2-74.8] | - | - | - | - | - | - | - | - |
| Spanish multilingual | - | - | - | - | 64.5 | [57.8-70.7] | 35.8 | [19.5-56.2] | - | - | - | - | - | - | - | - |
| Indigenous only | - | - | - | - | 15 | [11.2-19.9] | 9.4 | [3.6-22.1] | - | - | - | - | - | - | - | - |
| Highest level of education attained | | |  |  |  |  |  |  |  |  |  |  |  |  |  |  |
| None | 15.8 | [13-19] | 8.9 | [6.4-12.2] | 35.5 | [31.3-39.9] | 17.3 | [11-26.2] | 8.8 | [6-12.7] | 6.5 | [4-10.5] | 9.6 | [5.6-16] | 9.4 | [5.5-15.9] |
| Primary | 49.9 | [43.4-56.4] | 58.4 | [53.5-63] | 51.1 | [48-54.3] | 55.6 | [46.1-64.6] | 74.2 | [69.5-78.4] | 71.6 | [66.1-76.5] | 32.7 | [23.4-43.5] | 47.7 | [39.6-55.8] |
| Secondary | 21 | [16.3-26.6] | 18.9 | [15.9-22.3] | 6.9 | [5.5-8.6] | 9.7 | [6.9-13.6] | 8.2 | [5.8-11.5] | 9.3 | [7-12.2] | 37.5 | [30.3-45.2] | 28.6 | [23.2-34.8] |
| High school or higher | 13.3 | [9.4-18.5] | 13.9 | [10.3-18.4] | 6.5 | [4.4-9.3] | 17.4 | [8.2-33.1] | 8.8 | [6.4-12] | 12.6 | [9.7-16.3] | 20.2 | [12.6-30.7] | 14.3 | [8.1-23.9] |
| Literacy |  |  |  |  |  |  |  |  |  |  |  |  |  |  |  |  |
| Cannot read at all | 17.3 | [14.2-20.9] | 13.7 | [10.7-17.4] | 38.1 | [33.8-42.6] | 20.8 | [13.4-30.9] | 14.8 | [11-19.6] | 14.3 | [10.3-19.6] | 7.5 | [4-13.6] | 12 | [7.4-18.7] |
| Able to read a portion | 18.7 | [16.3-21.4] | 18 | [14.4-22.3] | 25.5 | [22.9-28.3] | 23.1 | [18-29.2] | 22.8 | [19.2-26.9] | 19.4 | [15.2-24.5] | 12.2 | [8.7-16.8] | 16 | [9.9-24.7] |
| Able to read | 64 | [58.6-69] | 68.3 | [62.8-73.3] | 36.5 | [32-41.1] | 56.1 | [44.1-67.3] | 62.4 | [56.2-68.3] | 66.3 | [60.3-71.8] | 80.3 | [71.6-86.8] | 72.1 | [61.3-80.8] |
| Marital status |  |  |  |  |  |  |  |  |  |  |  |  |  |  |  |  |
| Single | 18.7 | [15.4-22.5] | 20 | [17.5-22.7] | 10.7 | [9-12.6] | 13.1 | [9.8-17.1] | 18.3 | [14.4-22.9] | 18.8 | [15.2-23] | 23.5 | [17.2-31.3] | 22.1 | [18.2-26.6] |
| Married | 34.2 | [31.2-37.4] | 29.6 | [26.8-32.6] | 33.7 | [30.1-37.6] | 52.4 | [43.3-61.4] | 32.3 | [28.2-36.6] | 29.2 | [25.5-33.3] | 35.9 | [30.1-42.1] | 26.1 | [21.8-30.8] |
| Domestic partnership | 42.3 | [38.8-45.8] | 47.3 | [43.7-50.9] | 50.3 | [46.2-54.5] | 30 | [24.1-36.7] | 45.5 | [41.5-49.6] | 48.7 | [43.9-53.5] | 35.5 | [29.7-41.9] | 49 | [43-55.1] |
| Other | 4.8 | [3.6-6.3] | 3.1 | [2.2-4.5] | 5.3 | [4.3-6.5] | 4.5 | [2.7-7.4] | 4 | [2.8-5.6] | 3.3 | [1.6-6.6] | 5.1 | [2.9-8.8] | 2.7 | [1.8-4.2] |
| Housewife | 84.2 | [79.6-88] | 82.2 | [77.8-85.9] | 93.5 | [91.4-95.1] | 81.7 | [66.9-90.8] | 90.2 | [87.3-92.5] | 86.7 | [80.6-91.1] | 75 | [66.2-82.1] | 78.5 | [70.8-84.6] |
| Owns a car or scooter | 12.8 | [10.3-15.9] | 17.1 | [14.3-20.4] | 9.9 | [7.9-12.4] | 15.8 | [10.1-23.9] | 15 | [12.2-18.3] | 14.2 | [11-18.2] | 13 | [8.1-20.1] | 19.8 | [14.9-25.7] |
| Experienced seizures before delivery | 3.3 | [2.6-4.2] | 6.4 | [4.7-8.6] | 4.6 | [3.5-6] | 6 | [3.9-9.1] | 4.3 | [3.4-5.6] | 10.4 | [7.1-15] | 1.9 | [0.9-3.9] | 3.1 | [1.9-5.1] |
| Stayed at a maternal home before delivery | - | - | - | - | - | - | - | - | - | - | - | - | 6.8 | [3.3-13.5] | 14.5 | [8.8-23.1] |

**Supplemental Table 2. Characteristics of women at follow-up in intervention and comparison groups, overall and by country**

|  | **All countries** | | | | **Guatemala** | | | | **Honduras** | | | | **Nicaragua** | | | |
| --- | --- | --- | --- | --- | --- | --- | --- | --- | --- | --- | --- | --- | --- | --- | --- | --- |
|  | **Intervention** | | **Comparison** | | **Intervention** | | **Comparison** | | **Intervention** | | **Comparison** | | **Intervention** | | **Comparison** | |
| *Observations* | *2409* |  | *865* |  | *874* |  | *345* |  | *687* |  | *192* |  | *848* |  | *328* |  |
|  | **%** | **95% CI** | **%** | **95% CI** | **%** | **95% CI** | **%** | **95% CI** | **%** | **95% CI** | **%** | **95% CI** | **%** | **95% CI** | **%** | **95% CI** |
| Age |  |  |  |  |  |  |  |  |  |  |  |  |  |  |  |  |
| 15-24 | 46 | [43.7-48.3] | 40.8 | [37.6-44.1] | 41.2 | [37.9-44.6] | 39.9 | [34.5-45.5] | 43.8 | [39.6-48.1] | 37.2 | [30.1-45] | 49.2 | [45.6-52.9] | 44.7 | [40.6-48.9] |
| 25-34 | 38.4 | [36-40.8] | 41.8 | [38.9-44.8] | 41.1 | [37.5-44.9] | 41.5 | [37-46.2] | 37.8 | [33-42.9] | 42.9 | [34.5-51.8] | 37 | [33.4-40.7] | 41.6 | [38.2-45.1] |
| 35-49 | 15.6 | [14-17.5] | 17.3 | [14.9-20] | 17.7 | [15.4-20.3] | 18.6 | [14.8-23.1] | 18.4 | [14.8-22.7] | 19.8 | [14.5-26.5] | 13.8 | [11.3-16.7] | 13.7 | [10.3-18] |
| Primiparous | 33.9 | [31.4-36.4] | 31.1 | [28.1-34.2] | 27.7 | [24.6-30.9] | 22.8 | [18.4-27.9] | 32.2 | [28.5-36] | 36.2 | [30.4-42.4] | 37.8 | [33.8-42] | 40.4 | [35-46] |
| Area of residence |  |  |  |  |  |  |  |  |  |  |  |  |  |  |  |  |
| Rural | 79.3 | [70.9-85.8] | 83.8 | [74.6-90.1] | 85.8 | [74.8-92.4] | 94.7 | [79.1-98.8] | 84 | [69.9-92.2] | 89.9 | [69.3-97.2] | 74.5 | [60.4-84.9] | 63.1 | [41-80.8] |
| Urban | 20.7 | [14.2-29.1] | 16.2 | [9.9-25.4] | 14.2 | [7.6-25.2] | 5.3 | [1.2-20.9] | 16 | [7.8-30.1] | 10.1 | [2.8-30.7] | 25.5 | [15.1-39.6] | 36.9 | [19.2-59] |
| Asset index |  |  |  |  |  |  |  |  |  |  |  |  |  |  |  |  |
| Low | 51.3 | [47.7-54.9] | 41.9 | [35.2-48.9] | 50.6 | [44.8-56.3] | 46 | [35.6-56.8] | 61.1 | [53.8-67.9] | 51.5 | [33.6-69] | 49.2 | [43.7-54.7] | 29.3 | [21.6-38.4] |
| Medium | 38.3 | [35.2-41.4] | 41.2 | [35.8-46.8] | 35.9 | [31.5-40.6] | 37.9 | [29.6-46.9] | 28.5 | [23.7-33.8] | 36.4 | [24.7-50] | 42.1 | [37.3-47] | 49.4 | [41.5-57.3] |
| High | 10.4 | [8.8-12.3] | 16.9 | [12.8-22.1] | 13.5 | [10.3-17.5] | 16.1 | [10.5-23.8] | 10.4 | [7.2-14.9] | 12.1 | [6.8-20.7] | 8.7 | [6.8-11.2] | 21.3 | [12.6-33.7] |
| Languages spoken (Guatemala) | |  |  |  |  |  |  |  |  |  |  |  |  |  |  |  |
| Spanish only | - | - | - | - | 20 | [13.3-28.9] | 56.3 | [43.8-68] | - | - | - | - | - | - | - | - |
| Spanish multilingual | - | - | - | - | 59.5 | [52-66.5] | 39 | [29.1-50] | - | - | - | - | - | - | - | - |
| Indigenous only | - | - | - | - | 20.5 | [15.8-26.3] | 4.7 | [1.9-11.3] | - | - | - | - | - | - | - | - |
| Highest level of education attained | | |  |  |  |  |  |  |  |  |  |  |  |  |  |  |
| None | 13 | [11.2-15.2] | 12.2 | [9.3-16] | 22.5 | [18.1-27.6] | 18.4 | [13.1-25.2] | 9.5 | [6.6-13.5] | 5.2 | [2.1-12.3] | 8.7 | [6.6-11.3] | 7.5 | [4.2-12.9] |
| Primary | 52.9 | [49.4-56.4] | 53 | [47.6-58.3] | 58.5 | [53.8-63.1] | 59.8 | [52.3-66.9] | 68.1 | [63.2-72.6] | 55.7 | [45.5-65.5] | 45.9 | [40.4-51.5] | 40.7 | [29.3-53.2] |
| Secondary | 23.2 | [20.6-26.2] | 19.7 | [16.2-23.7] | 10.7 | [7.9-14.2] | 12.9 | [8.6-18.8] | 12.6 | [9-17.3] | 21.9 | [16.4-28.7] | 33 | [28.7-37.5] | 28.6 | [20.8-37.9] |
| High school or higher | 10.8 | [8.7-13.4] | 15.1 | [11-20.4] | 8.3 | [5.6-12.1] | 8.9 | [4.9-15.8] | 9.9 | [6.9-13.9] | 17.2 | [10.2-27.4] | 12.5 | [9.1-16.9] | 23.2 | [13.4-37.1] |
| Literacy |  |  |  |  |  |  |  |  |  |  |  |  |  |  |  |  |
| Cannot read at all | 15.7 | [13.6-18.2] | 15.9 | [12.4-20.2] | 25.6 | [20.8-31.1] | 22.3 | [16.1-30] | 12.8 | [9.2-17.4] | 9.6 | [4.8-18.1] | 11 | [8.5-14.2] | 10.4 | [5.7-18.3] |
| Able to read a portion | 16.1 | [14-18.5] | 15.6 | [11.6-20.6] | 20.4 | [16.8-24.6] | 21.9 | [14.7-31.5] | 12.9 | [10-16.5] | 8.6 | [3.9-17.8] | 14.6 | [11.4-18.4] | 10.5 | [6.9-15.8] |
| Able to read | 68.1 | [64.7-71.4] | 68.5 | [61.6-74.6] | 54 | [47.6-60.3] | 55.8 | [44-66.9] | 74.3 | [67.5-80.1] | 81.8 | [68.7-90.2] | 74.4 | [69.5-78.8] | 79.1 | [69.7-86.1] |
| Marital status |  |  |  |  |  |  |  |  |  |  |  |  |  |  |  |  |
| Single | 6.9 | [5.7-8.4] | 8.6 | [6.5-11.3] | 5.9 | [4.4-7.7] | 5.2 | [3.3-8.2] | 11.3 | [8.8-14.3] | 17.7 | [11.6-26] | 6.4 | [4.5-9.1] | 7.8 | [4.9-12.2] |
| Married | 29.1 | [26.5-31.8] | 34.6 | [30.6-38.9] | 26.9 | [23.2-30.9] | 42.3 | [35.9-48.9] | 27.1 | [22-32.7] | 24 | [17-32.7] | 30.9 | [26.9-35.2] | 30.1 | [23.4-37.7] |
| Domestic partnership | 54.8 | [51.8-57.8] | 50.8 | [46.5-55.1] | 62.4 | [57.6-67] | 49.7 | [42.9-56.4] | 58 | [53-62.9] | 55.1 | [45.1-64.7] | 49.8 | [45.2-54.4] | 49.8 | [42.6-56.9] |
| Other | 9.1 | [7.5-11] | 5.9 | [4.5-7.7] | 4.8 | [3.4-6.7] | 2.8 | [1.6-4.9] | 3.7 | [2.5-5.4] | 3.3 | [1-9.8] | 12.9 | [10.2-16.1] | 12.3 | [9.1-16.6] |
| Housewife | 86.9 | [84.2-89.1] | 84.4 | [80.2-87.8] | 90 | [86.1-92.9] | 88.7 | [82-93.1] | 89.8 | [86-92.7] | 84.3 | [74.5-90.8] | 84.3 | [80-87.9] | 77.8 | [68.7-84.9] |
| Owns a car or scooter | 16.1 | [14-18.6] | 22.4 | [17.9-27.7] | 15.4 | [12.3-19.2] | 18.4 | [12.3-26.7] | 13.9 | [10-18.9] | 22.1 | [13.3-34.5] | 17.1 | [13.8-21] | 28.8 | [20.1-39.3] |
| Attended any antenatal care | 92.2 | [89.9-94.1] | 89.9 | [85.8-93] | 90.4 | [87.7-92.5] | 81.8 | [73.7-87.8] | 97.8 | [96.1-98.7] | 96.3 | [90.9-98.5] | 91.8 | [87.6-94.7] | 98.2 | [94.9-99.4] |
| Counseled regarding facility delivery | 68 | [64.6-71.1] | 64.5 | [58.2-70.3] | 36.8 | [31.5-42.4] | 42.2 | [32.7-52.4] | 88.8 | [85.2-91.7] | 85.8 | [76.3-91.8] | 79.9 | [74.9-84.1] | 84.3 | [75.2-90.5] |
| Advised to give birth in a facility | 71.2 | [67.6-74.5] | 67.1 | [60.8-72.8] | 41.7 | [36.3-47.2] | 44.6 | [34.6-55.1] | 89.2 | [86.4-91.5] | 86.5 | [80.7-90.7] | 83 | [77.1-87.6] | 88.5 | [81.9-92.9] |
| Informed should have a C-section | 35.6 | [32.1-39.2] | 32.6 | [27-38.8] | 14.8 | [11.7-18.7] | 16.7 | [10.8-24.9] | 51.7 | [42.8-60.6] | 50.4 | [35.2-65.6] | 43 | [37.5-48.7] | 45.1 | [35.8-54.6] |
| Advised to create a transportation plan | 35 | [31.8-38.2] | 35.1 | [29.6-41] | 18.1 | [14.1-22.8] | 23.4 | [17-31.4] | 62.4 | [54-70.1] | 51.8 | [35.3-67.9] | 37.3 | [32.7-42.2] | 41.8 | [34-50] |
| Experienced seizures before delivery | 3.3 | [2.6-4.2] | 2.6 | [1.5-4.5] | 4.8 | [3.3-6.9] | 0.8 | [0.2-2.4] | 3.3 | [2.1-5.1] | 6.4 | [2.7-14.5] | 2.4 | [1.6-3.6] | 2.8 | [1.2-6.3] |
| Stayed at a maternal home before delivery - | | | - | - | - | - | - | - | - | - | - | - | 26.2 | [19.6-34] | 20.9 | [14-30.2] |

**Supplemental Table 3: Unweighted OLS models predicting facility score** ^ⴕ^**, overall and by country**

|  | **All** | |  | **Guatemala** | **Honduras** | | **Nicaragua** | |
| --- | --- | --- | --- | --- | --- | --- | --- | --- |
|  | $\beta$ | **95% CI** | $\beta$ | **95% CI** | $\beta$ | **95% CI** | $\beta$ | **95% CI** |
| Essential Obstetric and Neonatal Care level | | |  |  |  |  |  |  |
| Basic | 0.000 | Ref. | 0.000 | Ref. | 0.000 | Ref. | 0.000 | Ref. |
| Comprehensive | 0.650*** | [0.26, 1.04] | 0.637* | [-0.12, 1.39] | 0.887*** | [0.28, 1.5] | 0.236 | [-0.53, 1.01] |
| Country |  |  |  |  |  |  |  |  |
| Guatemala | 0.000 | Ref. | - | - | - | - | - | - |
| Honduras | -0.299 | [-0.71, 0.11] | - | - | - | - | - | - |
| Nicaragua | -0.523** | [-0.97, -0.08] | - | - | - | - | - | - |
| Round |  |  |  |  |  |  |  |  |
| Baseline | 0.000 | Ref. | 0.000 | Ref. | 0.000 | Ref. | 0.000 | Ref. |
| Follow-up | 0.780** | [0.17, 1.39] | 0.714 | [-0.37, 1.8] | 1.053** | [0.15, 1.95] | -0.745 | [-2.76, 1.27] |
| Arm |  |  |  |  |  |  |  |  |
| Comparison | 0.000 | Ref. | 0.000 | Ref. | 0.000 | Ref. | 0.000 | Ref. |
| Intervention | 0.248 | [-0.32, 0.81] | 0.008 | [-0.91, 0.92] | 0.605 | [-0.26, 1.47] | -0.963 | [-2.95, 1.02] |
| Interaction: Follow-up x intervention | 0.132 | [-0.61, 0.87] | 0.532 | [-0.76, 1.82] | -0.553 | [-1.77, 0.66] | 1.540 | [-0.65, 3.73] |
| *Observations* | *137* | |  | *49* | *51* | | *37* | |
| ** p<0.1, ** p<0.05, *** p<0.01*  ⴕ ***Facility score*** *is a* *6-point score of the capacity to attend normal deliveries that included round-the-clock availability of skilled birth attendants, availability of basic equipment for antenatal and postpartum care (exam table, lamp, tape measure, sphygmomanometer, and stethoscope), and availability of basic inputs for delivery care such as oxytocin, methylergometrine or ergometrine maleate, and Ringer’s lactate/Hartmann’s solution or saline solution.* | | | | | | | | |

**Supplemental Table 4. Results of medical record review of uncomplicated deliveries in the last two years at baseline and follow-up for intervention and comparison groups by country**

|  |  | **Baseline, intervention** | | | **Follow-up, intervention** | |  | **Baseline, comparison** | |  | **Follow-up, comparison** | |
| --- | --- | --- | --- | --- | --- | --- | --- | --- | --- | --- | --- | --- |
|  | **N** | **%** | **95% CI** | **N** | **%** | **95% CI** | **N** | **%** | **95% CI** | **N** | **%** | **95% CI** |
| **Guatemala** |  |  |  |  |  |  |  |  |  |  |  |  |
| Uterotonic administered | 248 | 79.8 | [74-85] | 368 | 98.4 | [96-99] | 73 | 97.3 | [90-100] | 199 | 100 | - |
| Postpartum checks to standard ⴕ | 158 | 14.6 | [9-21] | 219 | 38 | [31-45] | 46 | 26.1 | [14-41] | 123 | 30.9 | [23-40] |
| **Honduras** |  |  |  |  |  |  |  |  |  |  |  |  |
| Uterotonic administered | 234 | 95.3 | [92-98] | 156 | 99.4 | [96-100] | 183 | 98.4 | [95-100] | 175 | 98.9 | [96-100] |
| Postpartum checks to standard ⴕ | 157 | 36.3 | [29-44] | 152 | 88.2 | [82-93] | 88 | 43.2 | [33-54] | 171 | 74.9 | [68-81] |
| **Nicaragua** |  |  |  |  |  |  |  |  |  |  |  |  |
| Uterotonic administered | 90 | 96.7 | [91-99] | 316 | 93.7 | [90-96] | 12 | 91.7 | [62-100] | 131 | 97.7 | [93-100] |
| Postpartum checks to standard ⴕ | 77 | 0 | [0-5] | 266 | 19.2 | [15-24] | 12 | 0 | [0-3] | 113 | 26.5 | [19-36] |
| ⴕ *Blood pressure and temperature checked four times during the first hour after delivery, twice during the second hour, and at discharge* | | | | | | | | | | | | |

**Supplemental Table 5. Reasons for not delivering in facility at baseline and follow-up in intervention and comparison groups by country**

|  |  |  |  | Culture, family, and beliefs ^a^ | | Finances and logistics ^b^ | | Health facility limitations ^c^ | | Other obstacles ^d^ | |
| --- | --- | --- | --- | --- | --- | --- | --- | --- | --- | --- | --- |
|  |  |  | *N* | % | 95% CI | % | 95% CI | % | 95% CI | % | 95% CI |
| Guatemala | Intervention | Baseline | *1805* | 60.9 | [56.6-65.2] | 16.2 | [13.0-19.5] | 9.2 | [6.2-12.2] | 21.7 | [17.8-25.5] |
|  |  | Follow-up | *653* | 80.3 | [75.6-85.0] | 13.2 | [8.9-17.5] | 10.4 | [7.4-13.4] | 10.1 | [6.6-13.6] |
|  | Comparison | Baseline | *303* | 49 | [33.7-64.2] | 15.3 | [6.3-24.2] | 6 | [0.8-11.3] | 31 | [19.3-42.7] |
|  |  | Follow-up | *247* | 86.1 | [80.8-91.4] | 13.8 | [7.7-19.9] | 3.4 | [0.8-6.1] | 8.9 | [3.8-14.1] |
| Honduras | Intervention | Baseline | *258* | 23.8 | [16.0-31.6] | 64.4 | [58.0-70.9] | 4.1 | [1.4-6.8] | 14.5 | [7.7-21.3] |
|  |  | Follow-up | *59* | 11.6 | [5.1-18.1] | 73.2 | [57.3-89.2] | 4.1 | [0-10.1] | 26.9 | [17.5-36.2] |
|  | Comparison | Baseline | *119* | 30.9 | [15.9-45.8] | 52.7 | [38.9-66.5] | 10.1 | [4.9-15.3] | 11.7 | [5.8-17.5] |
|  |  | Follow-up | *7* | 0 | - | 38.4 | [21.6-55.2] | 10.6 | [0-21.2] | 24.4 | [6.8-42.0] |
| Nicaragua | Intervention | Baseline | *49* | 59.2 | [43.9-74.4] | 31.1 | [19.3-42.8] | 11.1 | [6.0-16.1] | 15.3 | [5.0-25.5] |
|  |  | Follow-up | *120* | 35.6 | [18.6-52.5] | 40.8 | [26.6-55.0] | 12.3 | [3.9-20.6] | 29.9 | [18.4-41.3] |
|  | Comparison | Baseline | *50* | 22.6 | [14.8-30.4] | 30 | [15.9-44.0] | 6.4 | [0-13.1] | 45.1 | [31.2-59.1] |
|  |  | Follow-up | *18* | 17.6 | [10.0-25.2] | 51.6 | [32.7-70.4] | 5.2 | [0-13.8] | 30.8 | [8.8-52.8] |

^a^ Includes preferring labor under the care of a traditional birth attendant; preferring to give birth in the family home or another house; religious or cultural beliefs; wanting a traditional birth attendant to accompany; being prevented from going by husband, partner, or another member of the family.

^b^ Includes transportation problem, travel times, facility too distant, problems finding transportation, lacking someone to travel with the woman, having no place to stay, did not know where to go, and health facility charges for delivery.

^c^ Includes problems with health facilities (not having sufficient drugs or ill-equipped, problems with staff (not staffed, staff not trusted, not well informed or difficult to deal with, being previously treated poorly by the health facility), and having care denied when they have tried to go to a health facility.

^d^ Includes not being advised to deliver in a health facility and other reasons.

Model adjusted for age, education, parity, urban residence, asset index, and maternal literacy.
